# Nutrition induced pleomorphism and budding mode of reproduction in *Deinococcus radiodurans*

**DOI:** 10.1186/1756-0500-2-123

**Published:** 2009-07-07

**Authors:** Hiren M Joshi, Rao S Toleti

**Affiliations:** 1 Biofouling & Biofilm Processes Section, Water & Steam Chemistry Division, Bhabha Atomic Research Centre Facilities, Kalpakkam - 603 102, Tamil Nadu, India

## Abstract

**Background:**

Morphological adaptation is an important biological function of a microorganism to cope with its environment. Pleomorphism (to exist in a number of morphological forms) took centre stage in many discussions wherein a bacterium exhibits morphological transition and altered mode of reproduction in response to an environmental condition.

**Findings:**

To strengthen the concept on pleomorphism in bacteria, we report here different cell morphologies of *Deinococcus radiodurans *in response to variation in nutrient concentration. From our studies we attempt primary evidence towards the presence of significant population of monomer cells of *D. radiodurans *in specific culture condition. In this report we also illustrate with scanning electron micrographs an unusual budding mode of reproduction in *D. radiodurans *which was not reported till date for this group of bacteria.

**Conclusion:**

In a holistic view the study reflects on bacterial shape (morphotypes) and the physiological adaptation to a particular nutrient environment. The discovery of budding mode of reproduction in *Deinococcus *will be of interest to microbiologists. It can serve as a model system to understand the mechanism of budding process at molecular level.

## Background

The *Deinococcaceae *are a family of non-spore-forming bacteria which exhibit extreme resistance to ionizing radiation and desiccation [1-3]. *D. radiodurans *strain R1 (the strain used in the present study) is the well characterized member of *Deinococcaceae *and exist as individual tetrads in its natural milieu [4]. During different growth phases, *D. radiodurans *predominantly exists as tetrads, with a fraction of population as aggregates of sextet or octets [5]. Daly et al., [6] observed that *D. radiodurans *grows in tryptone, glucose and yeast extract (TGY) medium as clusters of two cells (diplococci) in early stages of growth and as four cells (tetracocci) in late growth phase. Venkateswaran et al., [2] demonstrated that *D. radiodurans *grows as diplococci in a defined minimal medium (DMM) which contains mixture of amino acids and other constituents. Chou & Tan [7] reported that the concentration of mono/divalent cations (Na, K, Li, Mg and Ca) plays a major role in multicell formation in *Deinococcus *and low salt medium such as TGY or nutrient broth also produce similar multicell forms in salt added media. In response to salt addition the diploid/tetrad (2/4) cells grew and divided without separation and formed 8, 16 and 32 cell units in succession [7]. This phenomenon of morphological transition is termed pleomorphism i.e., to exist in a number of morphological forms or morphotypes in response to environmental conditions [8].

## Methods

In this investigation we have grown *D. radiodurans *exclusively in TGY medium and observed different morphotypes of the bacterium by varying the culture medium concentration. During the study *Deinococcus *cells were harvested in the late log phase (48 hrs growth) and subjected to microscopic observation. To avoid any fundamental bias in determining bacterial shape and size measurements, we used confocal scanning laser microscopy, scanning electron microscopy and dynamic light scattering techniques for determination of the shape and size of the bacterium.

### Culture conditions

The strain of *Deinococcus radiodurans *R1 (B 2906, NRRLB) was maintained on TGY (tryptone-glucose-yeast extract) medium consisting of 5 g tryptone, 3 g yeast extract, 1 g glucose and 1.5% agar was added to prepare solid medium for sub-culturing and culture purity study. Phosphate buffered saline [PBS] at pH 7.2 was prepared using dehydrated phosphate buffer powder (BBL, USA) and 0.85% NaCl as per the manufacturer protocol.

### Growth conditions

The inoculated TGY broth cultures were incubated at 30°C in an orbital shaker at 100 rpm. The late log phase culture (48 hr of incubation) was centrifuged at 8,000 rpm for 5 minutes at 20°C. After two repeated washing with PBS, the bacterial cells were harvested, and used for further experimentation.

### Dynamic Light Scattering (DLS)

DLS measurements on dilute suspensions of *D. radiodurans *were performed using a Malvern 4700 (Malvern Instruments, UK) light scattering unit equipped with a Malvern made goniometer, a multi-tau correlator, and a 2.5 W (Ar^+ ^+ Kr^+^) ion laser (Spectra-Physics (Mountain View, CA, model No. 2018-RM) operating at 514.5 nm. In DLS measurements, the incident laser beam was vertically polarized and the scattered intensity was collected using a Glan-Thompson prism (Melles Griot, Rochester, NY), placed in front of the PMT in the vertical and horizontal polarization respectively. For DLS measurements, the cell pellet was further washed twice with 0.2 μm filtered PBS buffer. The washed pellet was resuspended directly into the light scattering cell (quartz) of 8 mm inner diameter with 1 ml of ultra-filtered PBS buffer. Further dilutions were made with the same ultra-pure buffer to the required concentrations.

### Confocal Scanning Laser Microscopy (CSLM)

After harvesting the bacterial cells were washed twice with PBS buffer and finally suspended in 1 ml of PBS. 50 μl of *D. radiodurans *suspension was spread on to a glass slide and air-dried. The smear was stained with 20 μl of 0.02% of acridine orange (absorption maximum; 490 nm, emission maximum; 520 nm) for a few minutes (Acridine orange is an intercalating dye that binds to the double stranded DNA inside the bacteria cells, and these dye-tagged molecules of double-stranded DNA emit fluorescence signal in green when excited with an Argon ion laser operating at 488 nm). Excess stain was washed with ultra-pure water, and the stained sample was air-dried. A thin cover slip was placed on the smear and the slide was mounted on to the microscope stage for observation. A confocal laser scanning microscope (Leica TCS SP2 AOBS) equipped with a Leica DMIRE2 inverted microscope (Leica Microsystems, Germany) was used to acquire images of *Deinococcus *bacteria. Water immersion objective (63 × 1.2 NA) was used for observing the bacterial smears. A 488 nm line of Argon laser was used for excitation of the acridine orange stained sample. Emission was collected in the bandwidth 515 to 540 nm to detect cells exhibiting fluorescence. All the images were taken by scanning a frame of 512 × 512 pixels with the laser beam in the x, y plane, and each image was averaged over 40 frames for a better signal/noise ratio.

### Scanning Electron Microscopy (SEM) Studies

A pure culture of *D. radiodurans *grown in 1:100 dilution TGY medium was processed to SEM analysis. The glutaraldehyde fixed cells were smeared on to glass slides and freeze dried. The specimens were later sputter coated with gold-palladium and observed in a Philips ESEM model XL30.

## Results

**Figure 1:**
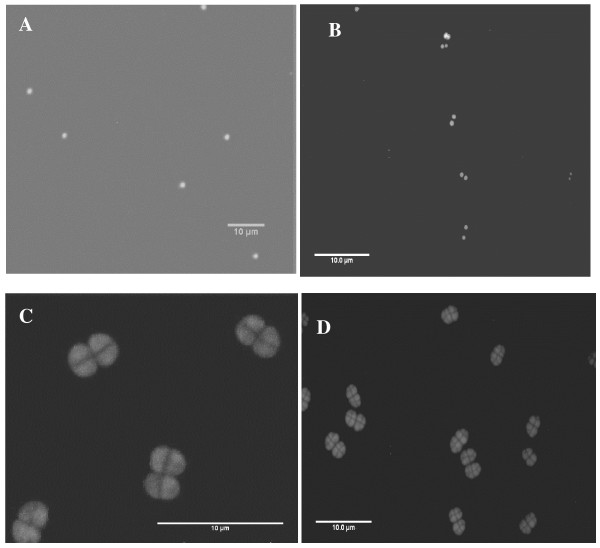
**Epi-fluorescence and confocal laser microscopic images of nutrition induced morphotypes of *D. radiodurans*, A – Monomer cells (CSLM image); B – Dimer cells (CSLM image); C – Tetramer cells (CSLM image); D – Multimer forms (Epi-fluorecscence image)**. Scale bar = 10 μm. Culture condition for A = 1: 100 dilution of TGY; B = 1:100; C = 1:10; D = without dilution.

**Figure 2:**
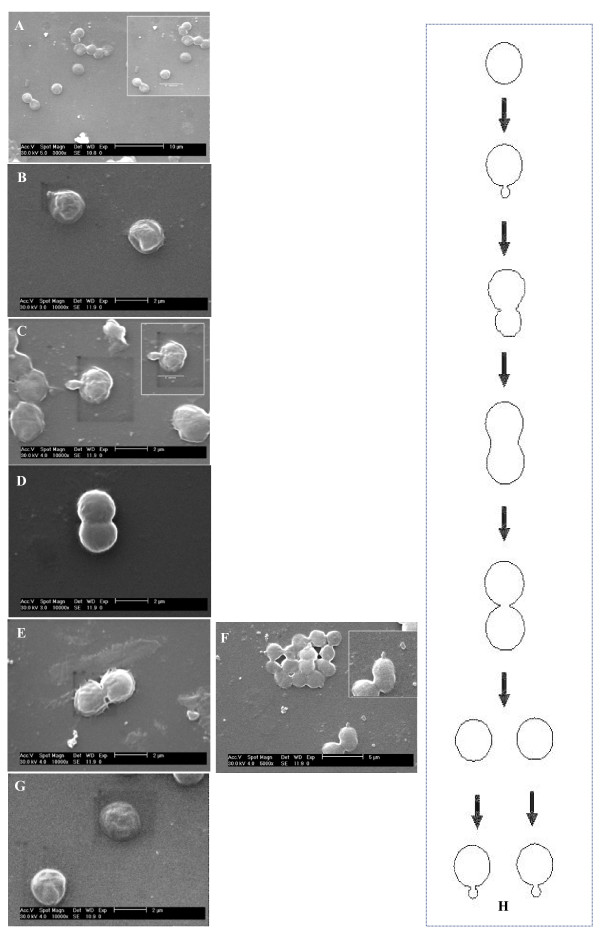
**SEM images of budding mode of reproduction in *D. radiodurans *along with schematic illustration**. A – Monomer cells of *D. radiodurans; *B – Bud initiation in monomer cell; C – Bud elongation stage; D – Mature bud stage; E – Constriction formation between mother and daughter cell; F – Bud initiation before separation of cell; G – Separated daughter cells. Panel H shows a schematic model for budding mode of reproduction. Culture condition 1: 100 dilution in TGY; Grown at 30°C for 48 hrs.

**Figure 3:**
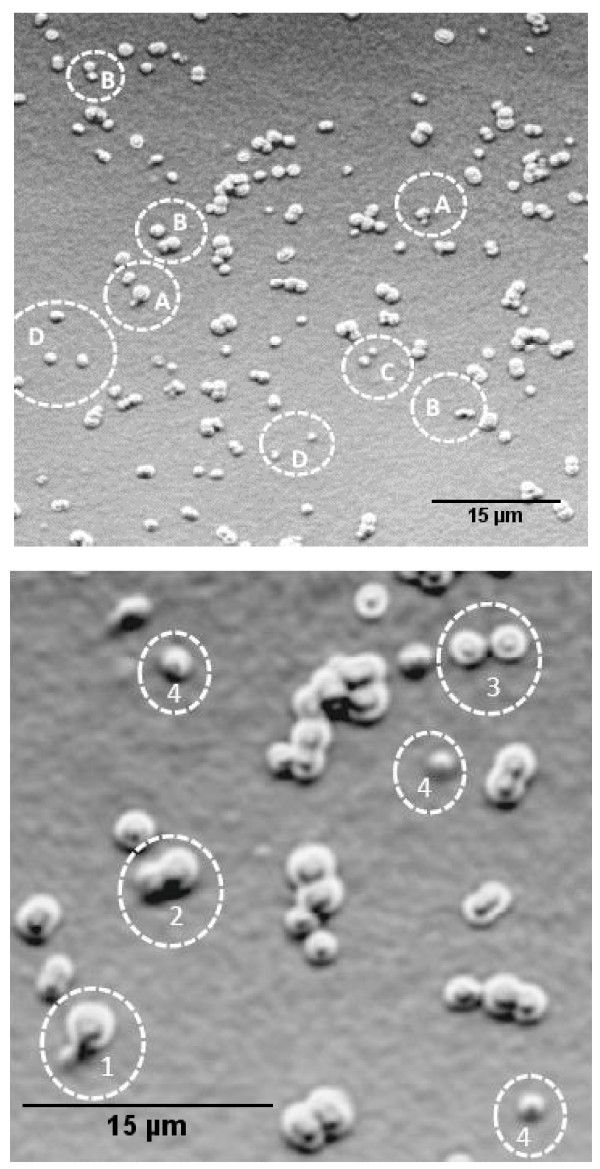
**a: CSLM transmission image showing budding in *Deinococcus radiodurans*: A – Bud formation, B – elongation of cell, C – Detachment of daughter cell, D – Monomer cells**. Culture condition 1: 100 dilution in TGY; Grown at 30°C for 48 hrs. **3b**: Magnified view of CSLM image: Different stages of cell division, 1 – bud initiation, 2 – elongation, 3 – two daughter cells, 4 – monomer cells. Culture condition 1: 100 dilution in TGY; Grown at 30°C for 48 hrs.

In this study we have grown *D. radiodurans *exclusively in TGY medium and observed different forms of the bacterium morphology by varying the culture medium nutrient concentration. Distinct *D. radiodurans *monomer cells (size 1.62 μm), diploid cells (size 1.96 μm) were observed at 1:100 dilution. Tetrads (size 2.05 μm) were detected at 1:10 dilution and multimer forms (size 3.45 μm) at full strength. Table 1 describes the size measurement values recorded from DLS and CSLM technique of different morphologies of *D. radiodurans*. DLS values obtained in an earlier study were also compared. Figure 1(A–D) describes the Confocal Laser Scanning Microscope (CSLM) images of monomer, diploid, tetrad and multimer forms of the *D. radiodurans*. In the highly diluted medium, we have noticed for the first time significant population of *D. radiodurans *monococcus cells (Figure 1A, 2A, G & Fig 3b). Notably, we also report for the first time on the budding mode of reproduction in *D. radiodurans *R1 species. SEM and CSLM images demonstrate morphological evidence of the unusual budding process in *D. radiodurans *which was illustrated in Figure 2(A–G), Figure 3a and 3b.

**Table 1:** Size distribution of different forms of *D. radiodurans *using Confocal Laser Scanning Microscopy and Dynamic Light Scattering.

***D. radiodurans *morphotype**	**CSLM**^a^	**DLS**^a^	**DLS**^b^
Monomer	1.62 ± 0.15	1.55 ± 0.02	ND

Dimer	1.96 ± 0.12	2.12 ± 0.04	2.06 ± 0.03

Tetramer	2.05 ± 0.08	2.32 ± 0.03	2.22 ± 0.02

Multimer	3.45 ± 0.12	3.55 ± 0.05	3.61 ± 0.04

a = present study; b = Jena et al., [9]; ND = not detected

## Discussion

Among the technologies currently being developed for treatment of toxic wastes bioremediation using extremely radiation-resistant organism, *Deinococcus radiodurans *shows promising application [1]. *Deinococcus radiodurans *is a non-pathogenic, desiccation-resistant, solvent-tolerant soil bacterium that can survive acute (short) exposure to an ionizing irradiation (~1.5 Mrads). This bacterium can also grow in the presence of chronic (continuous) gamma irradiation [4]. The *Deinococcus radiodurans *cells are usually spherical in nature, dividing in pairs and are non- motile [9]. *Deinococcus radiodurans *generally exist as tetrads, with fraction of population as sextet or octet in TGY (tryptone-glucose-yeast extract) medium [9]. There were reports that *D. radiodurans *can grow in multimeric forms under the influence of salt concentration or as diploid cells in defined minimal media (DMM) [4,7]. This report illustrates significant presence of *D. radiodurans *monococcus cells at low nutrient concentrations (Figure 1A, 2A, G & Figure 3b). This unusual observation of monomeric units of *D. radiodurans *contradicts the findings of Daly et al., [6] who reported that *D. radiodurans *does not grow as monomers and survival of single cell population cannot be determined experimentally. However, Oyazu et al., [10] reported that *Deinococcus grandis *grows as single cells in either TGY or DMM. The observations from the present data demonstrate that using different concentrations/dilutions of the basic medium TGY one can decipher various morphologies of *D. radiodurans*. We attribute this phenomenon (i.e., variation in morphology) to nutrition induced pleomorphism. The observed pleomorphism in *D. radiodurans *implies that, limitations on the sizes of a bacterium are not due to the ability to take up nutrients *per se *but arise from the competition for available nutrients as described by Young [11]. Nutrient concentration, chiefly results in formation of smaller, faster growing cells like monomer and dimer forms compared to larger size (tetrads or multimers). The presence of monomer or dimer units is the result of size oriented higher surface to volume ratio, which aid in nutrient scavenging in a deficient environment. In effect a bacterium can enhance its survival by simply adapting its morphological forms to propagate in a particular environment [12]. Although, there are many established reports indicating pleomorphism in different species of bacteria [12-14] still the phenomena is debated. Young, [11] suggested that, by accruing evidence for cell shape, it is possible to manipulate bacterial morphology with enough confidence. Concurrently, one can also arrive at how morphological changes affect survival in different nutrient deficit conditions. This investigation divulges with experimental evidence that cell shape *per se *is influenced by nutrient acquisition property. The study also provides impetus for further experimentation in varied nutritional situations which facilitate favourable conditions for one bacterial shape over another. This report concludes that nutrient concentration in a culture media of *D. radiodurans *incites pleomorphism and we support this general concept in bacteria.

Generally, bacteria follow simple binary fission for propagation but unusual reproductive ways such as budding is also exhibited in some cases [15]. For example the filamentous *Bacillus *sp., strain NAF 001 exhibits budding growth [16], which is not culture-dependent. However, in the present study we consider the budding process in *D. radiodurans *is culture condition dependent. The dilute nutrient environment could have resulted in the monomer forms, consequently resulting in the bud formation (Figure 2A–G). Commonly, budding results in formation of smaller daughter cell which subsequently detaches and grow independently. We conclude that the dilute nutrient condition could also have induced budding mode of reproduction in *D. radiodurans*.

This study has significant ramifications primarily on growth and reproduction of *Deinococcus *and related bacteria. In a holistic view it reflects on bacterial shape (morphotypes) and the physiological adaptation to a particular environment. The conflicting aspect of the true morphology of the bacterium in natural environment and observed morphology in laboratory conditions always posed questions to microbiologists. It can be surmised that the dilute nutrient condition of the culture broth represents the natural nutrient environment of the *D. radiodurans *which is largely nutrient depleted [10]. A recent report [17] describes that *Deinococcus *sp could be isolated only in ten times diluted culture media instead of full strength medium, this observation also corroborates our findings. Similarly, we conjecture that nutrient starvation conditions can result in cell asymmetry and further budding type of cell division. This phenomenon of nutrient depletion seems to be analogous to the spore formation process of *Bacillus subtilis*, wherein nutrient limitation leads to formation of cellular asymmetry and spore formation. The discovery of budding mode of reproduction in *Deinococcus *will be of great interest to microbiologists. It can serve as a model system to understand the mechanism of budding at molecular level particularly in case of some extremophiles.

## Competing interests

The authors declare that they have no competing interests.

## Authors' contributions

Both the authors contributed to the design of the study and interpretation of data. JHM contributed to basic microbiology work and carried out confocal microscopy imaging. TSR contributed to SEM analysis and drafted the manuscript. All authors read and approve the final manuscript.
